# Annual decline in forced expiratory volume and airway inflammatory cells and mediators in a general population-based sample

**DOI:** 10.1186/s12890-018-0765-7

**Published:** 2019-05-09

**Authors:** Natalia Kononova, Liv Ingunn Bjoner Sikkeland, Faiza Mahmood, Maria Vistnes, Johny Kongerud, Gunnar Einvik, Vidar Søyseth

**Affiliations:** 1 Faculty of Medicine, Institute of Clinical Medicine, University of Oslo, Oslo, Norway; 2 Department of Respiratory Medicine, Oslo University Hospital, Oslo, Norway; 3 Unit of Medical Biochemistry, Division of Diagnostics and Technology, Akershus University Hospital, Lørenskog, Norway; 4 Department of Internal Medicine, Diakonhjemmet Hospital, Oslo, Norway; 5 Institute for Experimental Medical Research, Oslo University Hospital, Ullevål, Oslo, Norway; 6 Department of Pulmonology, Division of Medicine, Akershus University Hospital, Lørenskog, Norway

**Keywords:** Healthy individuals, Annual decline in forced expiratory volume, Airway inflammation, Induced sputum, Sputum cytokines and chemokines, Sputum cells

## Abstract

**Background:**

Few studies have examined the relationships between sputum inflammatory markers and subsequent annual decline in forced expiratory volume in 1 s (dFEV_1_). This study investigated whether indices of airway inflammation are predictors of dFEV_1_ in a general population-based sample.

**Methods:**

The study, conducted from 2003 to 2005, included 120 healthy Norwegian subjects aged 40 to 70 years old. At baseline, the participants completed a self-administered respiratory questionnaire and underwent a clinical examination that included spirometry, venous blood sampling, and induced sputum examination. From 2015 to 2016, 62 (52%) participants agreed to a follow-up examination that did not include induced sputum examination. Those with a FEV_1_/forced vital capacity (FVC) ratio <  0.70 underwent a bronchial reversibility test. The levels of cytokines, pro-inflammatory M1 macrophage phenotypes were measured in induced sputum using bead-based multiplex analysis. The associations between cytokine levels and dFEV_1_ were then analysed.

**Results:**

The mean dFEV_1_ was 32.9 ml/year (standard deviation 26.3). We found no associations between dFEV_1_ and the baseline indices of sputum inflammation. Seven participants had irreversible airflow limitation at follow-up. They had lower FEV1 and gas diffusion at baseline compared with the remaining subjects. Moreover, two of these individuals had a positive reversibility test and sputum eosinophilia at baseline.

**Conclusions:**

In this cohort of presumably healthy subjects, we found no associations between sputum inflammatory cells or mediators and dFEV_1_ during 10 years of follow-up.

**Electronic supplementary material:**

The online version of this article (10.1186/s12890-018-0765-7) contains supplementary material, which is available to authorized users.

## Introduction

Chronic obstructive pulmonary disease (COPD) is characterized by reduced forced expiratory volume in 1 s (FEV_1_) and is associated with an enhanced inflammatory response in the airways and the lungs [1]. Environmental factors can contribute to airway inflammation, including tobacco smoke, occupational agents, urban air pollutants, and fuel wood [2]. There is evidence that exposure for air pollution is associated with impairment of lung growth and increased risk of reduced adult lung function. This is shown by a study from Los Angeles region that investigated the percentage of 18-year-old participants with attained FEV_1_ below 80% of the predicted value. This percentage turned out to be much higher in the more polluted zones as compared to the less polluted zones [3].

Several types of cells are involved in COPD inflammation, including macrophages, epithelial cells, dendritic cells, neutrophils, eosinophils, and T and B lymphocytes, and higher levels of these cells in the lungs are associated with higher levels of inflammation and with disease progression. There are studies describing normal sputum composition. Induced sputum from healthy subjects is rich in macrophages and neutrophils and poor in the eosinophils, lymphocytes and epithelial cells and number of macrophages is higher than neutrophils. The relative number of neutrophils compared with macrophages increases with COPD developing and aging process [4–7].

The eosinophilic phenotype of COPD shares some features with asthma, such as the reversibility of airway obstruction and a greater response to corticosteroids than other types of COPD [8]. Inflammatory cells release inflammatory mediators that contribute to the pathophysiology of the disease, including cytokines, chemokines, lipid mediators, and growth factors [10]. Cytokines reflect both T helper 1 (IFN-γ), T helper 2 (IL-6), and T helper 17 (IL-17a, IL-17f, IL-23) responses and the activation of pro-inflammatory M1 macrophage phenotypes (CCL2, CCL3, CCL4, CXCL8). Notably, these cytokines play central roles in the pathogenesis of COPD. Activated murine M1 macrophages contribute to the pathology of the disease by releasing tissue-destructive enzymes that can cause lung destruction and airway damage. T helper 1 and T helper 2 cells are involved in hyper mucous secretion [9, 10].

Airway inflammation can be examined using a non-invasive procedure that involves sputum collection after the inhalation of a hypertonic salt solution. This test has been standardized and is used as a diagnostic tool to research airway diseases such as asthma and COPD [11]. In a longitudinal study of healthy individuals, Fletcher and co-workers showed that subjects who developed COPD had accelerated decline in FEV1 [12] compared to individuals who did not develop COPD. This hallmark study also found that dFEV_1_ values were higher in smokers than in non-smokers and that smoking cessation normalized dFEV_1_ [13]. Newer studies suggested that only 50% of adult smokers developed reduced lung function because of accelerated fall in FEV1 another part never archive the normal level of lung function in early adulthood [14].

Some retrospective studies have examined bronchial inflammation by collecting induced sputum from healthy individuals and investigating the associations between bronchial inflammation and systemic inflammation and dFEV_1_. These studies found that neutrophils are elevated in the sputum of smelter workers and healthy smokers and that rapid dFEV_1_ is associated with an increased level of sputum neutrophilia [15, 16]. However, only a few studies have prospectively investigated the association between dFEV_1_ and indices of airway inflammation [17, 18]. As sputum induction is time consuming, these studies only included a few participants, and more studies are needed.

The current prospective study collected sputum data from 120 randomly selected healthy subjects living in the Akershus University Hospital catchment area in Norway who were followed-up for 10 years. Of the 120 subjects, 62 were available for re-examination. We hypothesized that in this population, inflammatory markers in sputum would predict increased decline in FEV1 or the development of COPD. The main aim of this study was to investigate the associations between airway inflammatory markers in induced sputum and subsequent dFEV_1_. A second aim was to investigate the relationship between inflammation at baseline and chronic airflow limitation at the end of the 10-year follow-up period.

## Methods

### Study design and subjects

**Fig. 1:**
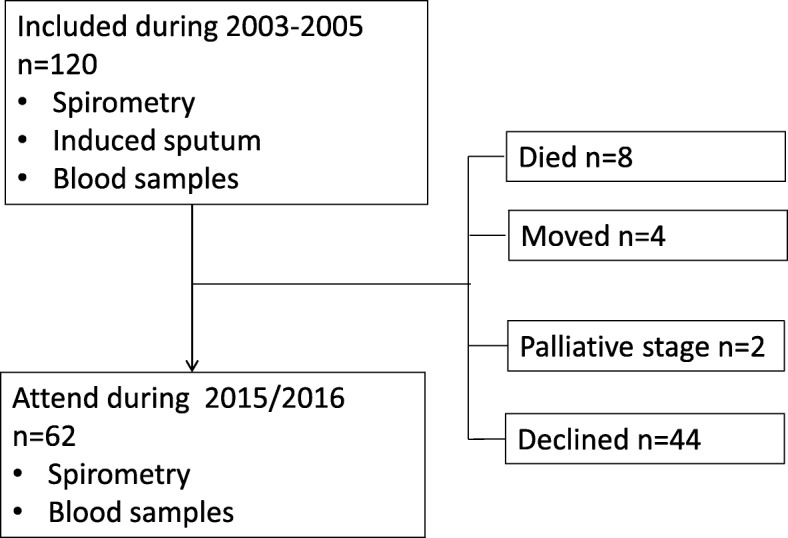
Flow chart of the participants in the study

A random sample of 120 apparently healthy individuals aged 40 to 70 years was drawn from the general population of Akershus County in South-Eastern Norway. These individuals were invited to participate in a baseline sputum examination in 2003 to 2005 and in a follow-up examination in 2015 to 2016. At the follow-up, 62 of the subjects (52%) were available for re-examination. A total of 44 participants declined to attend the follow-up visit, while the remaining 14 subjects were not available for other reasons: 8 participants died during follow-up, 4 moved outside the catchment area, and 2 were in palliative care due to cancer colon (*n* = 1) and multiple myeloma (n = 1) (Fig. 1).

At follow-up, all participants completed a self-administered questionnaire about their respiratory symptoms, smoking habits, medical history, and medical treatment (Additional file 1). Former smokers had to be quitters for at least one year or more. Pack-years were calculated by multiplying the number of packs of cigarettes smoked per day by the number of years the person has smoked.

They also underwent a clinical examination that included spirometry and venous blood sampling at baseline and at re-examination (Fig. 1 and Table 1). 56 subjects accepted a reversibility test. Induced sputum was collected only at baseline.

**Table 1:** Demographic data at baseline according to participation in follow-up

	Total	Follow-up visit		*P*-value
	*n* = 120	Yes (*n* = 62)	No *(n* = 58)	
Demographic data				
Male, *n* (%)	61 (51)	35 (57)	26 (45)	0.20
Age, years, mean (SD)	54.9 (9.7)	55.8 (9.5)	53.9 (9.8)	0.12
Height, m, mean (SD)	1.74 (0.1)	1.75 (0.1)	1.72 (0.11)	0.18
Weight, kg, mean (SD)	79.7 (16.6)	81.6 (15.1)	77.6 (18.2)	0.30
BMI, kg/m^2^, mean (SD)	26.2 (4.3)	26.5 (4.0)	26.0 (4.6)	0.35
Smoking history				
Pack-years, mean (SD)	8.1 (12)	9.9 (14.4)	6.2 (8.6)	0.10
Current, *n* (%)	25 (21)	10 (17)	15 (28)	0.15
Quitters, *n* (%)	29 (24)	18 (30)	11(20)	0.24
Never smokers, *n* (%)	60 (50)	32 (53)	28 (48)	0.87
Unknown, *n* (%)	6 (5)	2 (3)	4 (7)	0.36
Spirometry, mean (SD)				
FEV_1_, % of predicted	102 (15)	101 (14)	102 (15)	0.74
FVC, l	4.2 (1.2)	4.3 (1.2)	4.2 (1.2)	0.49
FVC, % of predicted	110 (14)	109 (15)	111 (14)	0.46

*SD* standard deviation, *BMI* body mass index, *FEV*_*1*_ forced expiratory volume in 1 s, *FVC* forced vital capacity. Student’s t-tests and chi-square tests were used to analyse differences between groups

### Spirometry and the reversibility test

Spirometry was performed as recommended by the European Respiratory Society (ERS) [19] in the respiratory laboratory at Akershus University Hospital. All participants performed a reversibility test with inhalation of 400 μg salbutamol at follow-up. The test was regarded as positive if FEV_1_ increased by at least 200 ml and 12% from the pre-bronchodilator (pre-BD) value. Participants who had a post-BD FEV_1_/FVC-ratio <  0.7 were considered to have post-BD airflow limitation (AFL).

The diffusion capacity of the lung for carbon monoxide (DLCO) was determined using the single-breath test for carbon monoxide as recommended by ATS/ERS. Reference values from a Norwegian cohort were used to calculate the prediction values [20].

### Induced sputum

Sputum cells were obtained using induced sputum as described elsewhere [21]. After baseline spirometry was performed, the participants inhaled isotonic saline (0.9%) for 7 min using an ultrasonic nebulizer (De Vilbiss, Sunrise Medical). The participants inhaled increasing concentrations (3, 4, 5%) of hypertonic saline until a sufficient amount of sputum was obtained. The total inhalation time was not recorded. Between every inhalation, the participants rinsed their nose and cleaned their upper airway before performing a “chest-type” cough, and sputum was collected and stored in a container placed on ice. Sputum was selected from saliva before the processing by selecting sputum plugs.

### Sputum processing and cell count

Sputum from each participant was processed within 2 h of collection. Sample volume and weight were recorded, then the sample was diluted with phosphate-buffered saline (PBS) containing 0.2% dithiothreitol (DTT) using a volume that was equivalent to 4 times the sample weight. This was homogenized by mixing for 10 min at room temperature, than the entire sample was filtered through a 48-μm pore mesh filter and centrifuged at 4 °C at 1000 rpm for 7 min. The supernatant was stored at − 80 °C until the cytokine analysis was performed. The total cell count was analysed using a haemocytometer counting chamber, and cell viability was determined using Trypan blue (0.4%, Sigma, St Louis, MO, USA). The sample was rejected if SQE contamination was 20% or more and we had to exclude 32% sputum samples.

### Cytospin preparation

The cell pellet was resuspended in PBS, and the cell concentration was adjusted to 1 × 10^6^ cells/ml. The cells were stained using Diff-Quick (Medion Diagnostics, Switzerland), a modified May-Grünwald-Giemsa method. The sample IDs were blinded, and the cells were counted by two independent researchers. If the standard deviations of the cell counts differed by 10% or more, the cells were counted twice by both researchers. At least 200 cells were counted for each cytospin. Visible squamous epithelial cells and bronchial epithelial cells were excluded from the cell count.

### Cytokine analyses

The concentrations of ten cytokines [IL-6, IL-23, IL-17A, IL-17F, IL-23, CXCL8, CCL2, macrophage inflammatory proteins CCL3 and CCL4, interferon-γ, and tumour necrosis factor-α (TNF-α)] were measured in the sputum samples using the Bio-Plex® protein array systems (Bio-Rad, Hercules, CA, USA), which are based on xMAP technology (Luminex, Austin, TX, USA). The analyses were performed according to the manufacturer’s protocol with minor modifications. Briefly, recombinant cytokines were reconstituted and serially diluted in PBS plus DTT to create a standard curve with a similar range as the samples. Beads with capturing antibodies plus standards or samples were added to 96-well microtiter plates and incubated for 2 h, then the detection antibody and streptavidin-PE were added. The plates were analysed with Bio-Plex®MAGPIX™ (Bio-Rad, Hercules, CA).

### Blood

Total and differential white blood cells were analysed by Sysmax XE-210008 within 48 h of blood collection into EDTA vacutainers.

### Statistics

Categorical variables and continuous variables were compared between groups using the chi-square test, student’s t-test, or one-way ANOVA (analysis of variance) as appropriate. Blood counts were log-transformed and all other data was normally distributed. The annual declines in forced vital capacity (dFVC) and in dFEV_1_ were calculated as the differences between the spirometry result at follow-up and baseline divided by the follow-up time in years. Values are reported as means (standard deviations). Two-sided *P*-values < 0.05 were considered significant.

Regression analyses was used for the multivariable analyses and all covariates were included together. Multivariable models were not adjusted for the other variables. The data were analysed using SPSS version 21.

## Results

The demographic data collected at baseline showed no significant differences between the follow-up participants, and those who did not participate in follow-up with regards to sex, age, or lung function (Table 1). The subjects in the follow-up group smoked more (9.9 pack-years), and there were fewer current smokers in the follow-up group than in the group that did not participate in follow-up*.* No one started to smoke during the observation period.

The mean dFEV_1_ was 32.9 (26.3) ml/year. Table 2 shows the distribution of dFEV_1_ values in tertiles according to demographic data, spirometry results, peripheral white blood cell count, and induced sputum cells using t-test.

**Table 2:** Annual declines in FEV_1_ (dFEV_1_) in tertiles according to relevant covariates at baseline

	Entire group *n* = 62	dFEV_1_, ml/year			*P*-value
		< 25 *n* = 20	25–38 *n* = 19	> 38 *n* = 23	
Demographic data					
Male, *n* (%)	35 (56,5)	9 (45)	10 (53)	16 (70)	0.25
Age, years, mean (SD)	55.6 (9.5)	54.5 (9.6)	56.4 (9.4)	55.7 (9.6)	0.80
Height, m, mean (SD)	1.75 (0.1)	1.72 (0.1)	1.77 (0.1)	1.76 (0.1)	0.36
BMI, kg/m^2^, mean (SD)	26.5 (4.0)	26.0 (3.4)	26.8 (4.8)	26.9 (3.8)	0.78
Smoking history					
Pack-years, mean (SD)	9.9 (14.4)	10.7 (14.4)	12.6 (15.8)	6.8 (12.2)	0.42
Current smoker, *n* (%)	10 (16)	4 (20)	4 (21)	2 (9)	0.49
Former smoker, *n* (%)	18 (29)	5 (25)	7 (37)	6 (26)	0.73
Never smoker, *n* (%)	32 (52)	10 (50)	8 (42)	14 (61)	0.39
Unknown, *n* (%)	2 (3)	1 (5)	0 (0)	1 (4)	0.63
Lung function data, mean (SD)					
dFEV_1_, ml/year	*32.9* (26.3)	4.3 (16.6)	33.0 (3.8)	62.5 (14.5)	–
FEV_1_, l, mean (SD)	3.27 (1.0)	3.1 (1.0)	3.2 (1.0)	3.5 (0.9)	0.47
FEV, % of predicted	102 (15)	100 (14)	98 (17)	104 (13)	0.38
FVC, l	4.3 (1.2)	4.1 (1.3)	4.3 (1.3)	4.6 (1.1)	0.49
FVC, % of predicted	110 (14)	109 (16)	107 (17)	111 (11)	0.67
FEV_1_/FVC, %	76 (6)	76 (6)	76 (7)	77 (6)	0.94
WBCs × 10^9^/l, GM (GSD)					
Total number	5.9 (1.7)	6.3 (1.3)	5.6 (1.8)	5.8 (1.8)	0.53
Neutrophils	3.4 (1.3)	3.7 (1.1)	3.2 (1.4)	3.3 (1.3)	0.43
Monocytes	0.4 (0.1)	0.4 (0.1)	0.4 (0.1)	0 (0.2)	0.35
Lymphocytes	1.8 (0.6)	1.8 (0.5)	1.8 (0.6)	1.8 (0.7)	1.00
Eosinophils	0.0 (0.1)	0.1 (0.1)	0 (0.1)	0.1 (0.1)	0.13
Sputum cells, %					
Macrophages	36 (17)	38 (20)	31 (18)	44 (12)	0.06
Neutrophils	61 (18)	60 (20)	67 (18)	53 (12)	0.05
Lymphocytes	2 (1)	2 (1)	2 (1)	2 (1)	0.63
Eosinophils	1 (2)	1 (1)	1 (1)	2 (4)	0.41

*SD* standard deviation, *FEV*_*1*_ forced expiratory volume in 1 s, *FVC* forced vital capacity, *GM* geometric mean, *GSD* geometric standard deviation, *WBCs* white blood cells. Blood counts were log-transformed and all other data was normally distributed. One-way ANOVA and chi-square tests were used to analyse differences between groups. Mean Packyear level is derived from both current and former smokers. Number of sputum samples was equal to number of participants in all three groups

We found no associations between dFEV_1_ and any of the covariates in Table 2. Surprisingly, the group with the largest dFEV_1_, which was > 38 ml/year, had more never smokers then the other groups, although there was no difference in age and sex distribution. Notably, 8 of the 10 participants who were current smokers at baseline quit smoking during the follow-up period.

We also investigated cytokine levels in the sputum. Table 3 shows the analysis of associations between dFEV_1_ and sputum cytokines and chemokines. We found no significant associations between sputum cytokines or chemokine levels and dFEV_1_ but correlation between log-transformed IL8 and log-transformed number of sputum neutrophils were highly significant (*p*-value < 0.001).

**Table 3:** Sputum cytokines and chemokines in tertiles of annual decline in FEV_1_ (dFEV_1_) in the entire cohort

	Total group	dFEV_1_ ml/year in tertiles			*P*-value
		< 25	25–38	> 38	
	*n* = 62	*n* = 20	*n* = 19	*n* = 23	
Cytokines and chemokines, GM (GSD)					
IL-8, pg/ml	711(2,8)	781(2.79)	698(3,8)	665(2.5)	0.79
IL-6, pg/ml	614 (22.8)	1002 (14.1)	604 (34.1)	406 (25.5)	0.65
IL-23, pg/ml	77.9 (8.1)	137 (6.7)	71.7 (7.9)	51.0 (8.8)	0.30
TNF-α, pg/ml	3.5 (7.5)	7.2 (5.6)	2.6 (7.9)	2.3 (8.4)	0.14
IFN-γ, pg/ml	49.3 (9.8)	108 (7.2)	38.3 (11.4)	30.8 (10.6)	0.17
IL-17a, pg/ml	426 (5.4)	865(15.0)	340 (7.4)	277 (5.4)	0.07
IL-17f, pg/ml	1203 (5.1)	2207 (3.9)	984 (5.6)	838 (5.4)	0.12
CXCL8, pg/ml	711 (2.8)	781 (2.7)	698 (3.4)	665 (2.6)	0.88
CCL2, pg/ml	63.7 (8.1)	89.6 (7.1)	68.5 (8.3)	44.7 (9.1)	0.55
CCL3, pg/ml	26.4 (8.4)	34.6 (10.1)	29.8 (10.1)	18.8 (6.1)	0.62
CCL4, pg/ml	50.5 (15.1)	57.1 (3.0)	63.0 (18.6)	37.9 (14.1)	0.81

*GM* geometric mean, *GSD* geometric standard deviation, *IL* interleukin, *TNF* tumour necrosis factor, *IFN* interferon, *CXCL* C-X-C motif ligand, *CCL* C-C motif ligand. Differences in means between groups were analysed using one-way ANOVA

We investigated the associations between dFEV1 as well as FEV1 at baseline as outcome variables, and IL-6, IL-8, IL-23, IL-17f, IL-17a, IFN-γ, TNF- α, CXCL8, CCL2, CCL3, CCL4 and sputum neutrophils as covariates. None of these relationships reached significant levels. It is likely that the variation in the outcome as well as explanatory variables were too wide, as the residuals of these regressions were normally distributed and in the normal range for FEV1 as well as dFEV1. During the follow-up period, 7 participants developed irreversible AFL. We compared the baseline data of these subjects with the data of participants who had no post-BD AFL (*n* = 49 subjects) (GOLD criterion). The groups showed no difference in sex distribution and only a negligible difference in dFEV_1_. The mean subject age was higher in the group with irreversible post-BD AFL than in those in the non-AFL group.

Table 4 shows that seven participants had post-BD AFL*.* We identified two distinct groups among the participants who had post-BD AFL: the first group of participants who had positive reversibility test (2 subjects) and the second group of participants, who did not respond to salbutamol (5 subjects)*.* Participants who had irreversible post-BD AFL at the end of follow-up had significantly lower predicted FEV_1_%, lower DLCO and KCO, higher peripheral white blood cell count (limited to neutrophils), and higher tobacco consumption at baseline compared with participants who had normal lung function at the end of follow-up (Table 4). The dFEV_1_ in first group was steeper than in the second group, but the FEV_1_/FVC ratio at baseline was similar in the two groups. The diffusion capacity of CO in the group with positive reversibility test was higher at baseline than in group without positive reversibility test and was equal to that of the healthy group. The baseline number of sputum eosinophils in subjects in the first group was higher than in subjects in the second group.

**Table 4:** Post-bronchodilator obstruction (post-BD FEV_1_/FVC-ratio < 70%) after inhalation of salbutamol 400 μg at the follow-up according to relevant characteristics at baseline

Baseline characteristics	Post-BD FEV_1_/FVC < 70%		*P*-val^1^
	No (n = 49)	Yes (*n* = 7)	
Spirometry, mean (SD)			
dFEV_1_, ml/year	34.5 (3.3)	42.5 (10.4)	0.42
FVC, l	4.2 (0.2)	4.2 (0.4)	0.62
FVC, % of predicted	108 (2)	106 (9)	0.70
FEV_1_, l	3.3 (0.1)	2.7 (0.3)	0.10
FEV_1_, % of predicted	84 (2)	70 (6)	< 0.01
FEV_1_/FVC%	78 (1)	66 (1)	< 0.01
DLCO, mmol/(min × kPa)	9.6 (0.3)	7.9 (0.9)	0.10
DLCO, % of predicted	100 (2)	83 (7)	< 0.01
KCO, mmol/(min × kPa × l)	1.7 (0.04)	1.3 (0.1)	< 0.01
KCO, % of predicted	120 (2)	102 (10)	0.01
Blood values			
Haemoglobin, g/dl	15.0 (0.16)	15.1 (0.6)	0.79
WBCs, 10^9^/l	5.9 (0.20)	7.7 (1.2)	< 0.01
Neutrophils, 10^9^/l	3.4 (0.7)	4.7 (0.8)	0.02
Eosinophils, 10^9^/l	0.1 (0.01)	0.2 (0.04)	0.38
Sputum cells			
Neutrophils, %	62 (3)	57 (7)	0.54
Macrophages, %	36 (3)	37 (5)	0.83
Eosinophils, %	0.60 (0.1)	3.7 (1.1)	< 0.01
Pack-years, mean (SD)	9.2 (1.7)	23.9 (1.4)	0.01
Age, years, mean (SD)	55.4 (1.3)	63.1 (2.5)	0.04
Sex, male, n (%)	27 (87)	4 (13)	0.83

*Neg-RT* negative reversibility test, *Pos-RT* positive reversibility test, *SD* standard deviation, *M (SE)* median (standard error), *CO* carbon monoxide, *DLCO* diffusion capacity in the lung for CO, *KCO* diffusion capacity in the lung for carbon monoxide per alveolar volume, *WBCs* white blood cells; *P*-val^1^, *P*-value for the comparison of post-BD AFL/Neg-RT and not obstructive; *P*-val^2^, ACO and not obstructive

## Discussion

The primary aim of this study was to test the hypothesis that airway inflammation in presumably healthy subjects is a determinant of accelerated dFEV_1_. We found no association between dFEV1 and inflammation at baseline. There was, however, a positive relationship between irreversible airflow limitation at the 10-year follow-up and sputum eosinophils at baseline, foremost in participants with the combination of airflow limitation and positive reversibility test.

Induced sputum testing is a safe, non-invasive procedure that can provide valuable information about the immune response in the central airways and in airway diseases [22]. However, it may be uncomfortable for the person being tested and is time-consuming. This may explain why few longitudinal studies have investigated the relationship of sputum and clinical outcomes. As early as 1996, Stanescu found that an accelerated annual decline in FEV_1_ correlated with the percentage of neutrophils and inversely correlated with the percentage of macrophages [16]. However, dFEV_1_ was analysed using variables obtained at follow-up rather than at baseline. Hence, the association between dFEV_1_ and sputum inflammation could be the result of, rather than a predictor of, the accelerated decline. More recent prospective studies are more in line with our study [17, 18]. Neither of these studies found any associations between inflammatory variables at baseline and subsequent dFEV.

Induced sputum has mostly been compared between healthy subjects (who are frequently young students) and patients with established clinical lung diseases, such as asthma or COPD. In this study, none of the subjects had diagnosed COPD at baseline. The participants lived in area without big industry and low air pollution. Accordingly, the study could only detect associations between dFEV_1_ and inflammatory indices in a healthy population. The number of sputum cells was in the same range as found in other sputum studies in general populations [7]. As the variability in cell counts was as high as 20%, sputum studies of dFEV_1_ in the general population should be considerably larger than this study.

There are several possible explanations for our negative findings. dFEV_1_ variability could be too small to measure. Notably, the mean dFEV_1_, as well as dFEV_1_ variation, was in the same range as found in other longitudinal studies of lung function in general populations [23, 24]. Moreover, some smokers may quit smoking during the follow-up, as in this study.

There were several interesting findings related to the second aim of the study. In particular, participants who had both post-BD AFL and a positive reversibility test at follow-up had significantly more sputum eosinophils at baseline than the other participants. Moreover, they had normal diffusion of CO at baseline and steeper dFEV_1_ than the other participants, although the latter relationship was not significant. Both of these observations are in line with other studies [25, 26]. Participants with post-BD AFL and positive reversibility test had a lower FEV_1_/FVC-ratio at baseline than those who had no post BD AFL at follow-up, but they were not older than the other participants. This was not in agreement with other studies [25].

The participants who had BD AFL and showed no reversibility with salbutamol at the follow-up had significantly lower diffusion of CO, higher peripheral white blood cell counts (neutrophils), higher cumulative consumption of tobacco, and they were older than the healthy group at baseline. All these findings are in agreement with other studies [25, 27] and indicate that these subjects had emphysema. However, they had roughly the same dFEV_1_ as those who had no post-BD AFL. This finding may be surprising, but recent studies have shown that COPD patients with low initial FEV_1_ may have dFEV_1_ that is within the normal range [28, 29].

The main limitation of this study was its small sample size, and therefore sensitive to clustering of the participants. However, the age and sex distribution of the sample was close to the target population. Hence, we do not believe that the sample was biased. However, as a sputum study this sample size should be sufficient to detect a clinically relevant relationship between airway inflammation and the decline in lung function. Sputum samples with more than 20% squamous cells (32%) were excluded insofar as sputum cells were included as explanatory variables. Several of the associations are interesting and merit further investigation in a larger cohort. In accordance with our results, such a prospective cohort include should more subjects with an increased risk of developing post-BD AFL or accelerated dFEV_1_. Regrettably, we were not able to identify participants with these risk profiles. Second, the total inhalation time varied between participants which are likely to have increased the variability of sputum neutrophil counts. The first portions produced are generally rich in neutrophils and later portions richer in macrophages. It could be one of the reasons why we did not find association between sputum cells and dFEV1. However, the total inhalation time was not recorded. Therefore, the lack of information on inhalation time should be considered as a limitation of the study.

Notably, DTT could influence the detection level of cytokines but it is difficult to say to what degree, and this fact could have impacted our results.

## Conclusion

In this cohort study of healthy subjects, we did not identify any inflammatory markers in sputum that predicted dFEV_1_ 10 years after the initial examination. However, we identified two distinct groups with post-BD AFL at the end of follow-up: one group with a positive reversibility test and another group with an irreversible BD test. The latter group had reduced DLCO and a history of much higher tobacco consumption than the other participants, a habit that suggests possible emphysema. The group with a positive reversibility test was characterized by having sputum eosinophilia at baseline. This study was relatively small, so this should be confirmed in a larger population.

## Additional file

Additional file 1: Questionnaire of clinical data. Questionnaire of clinical data was developed by research group at Pulmonary department Akershus University Hospital, Oslo, Norway and contains questions about respiratory symptoms, smoking habits, medical history and medical treatment. (PDF 99 kb)

## Abbreviations

ACO: asthma-COPD overlap syndrome
AFL: airflow limitation
BMI: body mass index
CCL: C-C motif ligand
CO: carbon monoxide
COPD: chronic obstructive pulmonary disease
CXCL: C-X-C motif ligand
dFEV_1_: annual decline in FEV_1_
dFVC: annual decline in FVC
DLCO: diffusing capacity of the lung for carbon monoxide
FEV_1_: forced expiratory volume in 1 s
FVC: forced vital capacity
GM: geometric mean
GSD: geometric standard deviation
Hb: haemoglobin
IFN: interferon
IL: interleukin
KCO: diffusing capacity of the lung for carbon monoxide per alveolar volume
Neg-RT: negative reversibility test
Pos-RT: positive reversibility test
Post-BD: post-bronchodilator airflow obstruction
SD: standard deviation
TNF: tumour necrosis factor
WBCs: white blood cells
