# Supplementary material for: Annual decline in forced expiratory volume and airway inflammatory cells and mediators in a general population-based sample
Source: BMC Pulm Med. 2019 May 9;19:90. doi: 10.1186/s12890-018-0765-7 (PMC6509765; doi:10.1186/s12890-018-0765-7)
Supplement: Supplementary file 1 — Questionnaire of clinical data. Questionnaire of clinical data was developed by research group at Pulmonary department Akershus University Hospital, Oslo, Norway and contains questions about respiratory symptoms, smoking habits, medical history and medical treatment. (PDF 99 kb) [file 12890_2018_765_MOESM1_ESM.pdf]

Questionnaire of clinical data

Smoking history:

1. Do you smoke or have you ever been smoking more than one cigarette a day during a year?

Yes No

2. If «yes « to question (1),
How many cigarettes did you smoke during this period of time (on average)?

………................ cigaretts / day

2.1 How old were you when you started to smoke regularly? ……… (years)

2.2 Have you ever tried to quit? Yes No

2.3 If you have quit smoking how old were you when you quit? ……… (years)

Respiratory symptoms:

1. Do you usually have any of the symptoms listed below even if you are not having cold?

1.1. Cough Yes No

1.2 Sputum Yes No

1.3 Piping in the chest Yes No

2. Have you ever been bothered by any of these symptoms in recent years?

2.1 Daily cough for at least three months a year during the last two years? Yes No

2.2 Dyspnea when you walk on a flat terrain or uphill? Yes No

2.2 Dyspnea when you walk at home on a flat surface? Yes No

3. Have you contacted a doctor because of any of these symptoms? Yes No

4. Do you still have these symptoms? Yes No

5. Have you received treatment with antibiotics ("penicillin") against pneumonia or bronchitis in the last three years? Yes No

If "yes" to question (5), how long ago did it happen?

0-3 months ☐

4-12 months ☐

1-2 years ☐

2-3 years ☐

More than 3 years ☐

<u>Diseases:</u>

Have you been diagnosed or treated for any of these diseases by a family doctor or at a hospital?

1. Angina pectoris (chest pain) Yes ☐ No ☐
2. Heart Infarction Yes ☐ No ☐
3. Heart surgery (bypass) Yes ☐ No ☐
4. Stenting to the heart Yes ☐ No ☐
5. Abdominal or thoracic aorta (the main blood vessel in the belly and thorax)? Yes ☐ No ☐
6. Blood vessels at the neck or legs Yes ☐ No ☐
7. High blood pressure Yes ☐ No ☐
8. Asthma Yes ☐ No ☐
9. COPD Yes ☐ No ☐
10. Chronic bronchitis Yes ☐ No ☐
11. Diabetes Yes ☐ No☐

<u>Medication:</u>

1. Do you use any of these drugs?

1.1 Aspirin Yes ☐ No ☐

1.2 Statins Yes ☐ No ☐

1.3 Inhaled steroids Yes □ No □

1.4 Other medications? Yes □ No □

…………………………………………………….

…………………………………………………….

<u>Education</u>

How many years of education do you have (including primary school)?……………………. years

Do you work? Yes □ No □

If "Yes" : How long have you worked in your current profession?

If ”No”: are you:
Student □ Retired □ Housewife □ Unemployed □ Disabled □

<u>Asbestos</u>

1. Have you ever worked with asbestos Yes □ No □

If “yes “,

1.1 How often did you work with asbestos?
Every day □ minimum 3 days/week □ Minimum 1-2 times a month □

less than once a month □

1.2 How many years have you worked with asbestos?………….. (years)
